# Measures of Appropriateness and Value for Breast Surgeons and Their Patients: The American Society of Breast Surgeons *Choosing Wisely*^*®*^ Initiative

**DOI:** 10.1245/s10434-016-5327-8

**Published:** 2016-06-22

**Authors:** Jeffrey Landercasper, Lisa Bailey, Tiffany S. Berry, Robert R. Buras, Amy C. Degnim, Oluwadamilola M. Fayanju, Joshua Froman, Jennifer Gass, Caprice Greenberg, Starr Koslow Mautner, Helen Krontiras, Roshni Rao, Michelle Sowden, Judy A. Tjoe, Barbara Wexelman, Lee Wilke, Steven L. Chen

**Affiliations:** 1 Gundersen Medical Foundation, La Crosse, WI USA; 2 Bay Area Breast Surgeons, Inc., Oakland, CA USA; 3 Norton Healthcare, Louisville, KY USA; 4 Anne Arundel Medical Center, Annapolis, MD USA; 5 Mayo Clinic, Rochester, MN USA; 6 MD Anderson, Houston, TX USA; 7 Mayo Clinic Health System, Owatonna, MN USA; 8 Women and Infants Hospital, Providence, RI USA; 9 University of Wisconsin School of Public Health and Medicine, Madison, WI USA; 10 Baptist Health South Florida, Miami, FL USA; 11 University of Alabama at Birmingham, Birmingham, AL USA; 12 University of Texas Southwestern Medical Center, Dallas, TX USA; 13 University of Vermont Medical Center, Burlington, VT USA; 14 Aurora Health Care, Milwaukee, WI USA; 15 Trihealth Cancer Institute, Cincinnati, OH USA; 16 University of Wisconsin of Madison, Madison, WI USA; 17 OasisMD, San Diego, CA USA

## Abstract

**Background:**

Current breast cancer care is based on high-level evidence from randomized, controlled trials. Despite these data, there continues to be variability of breast cancer care, including overutilization of some tests and operations. To reduce overutilization, the American Board of Internal Medicine *Choosing Wisely*^®^ Campaign recommends that professional organizations provide patients and providers with a list of care practices that may not be necessary. Shared decision making regarding these services is encouraged.

**Methods:**

The Patient Safety and Quality Committee of the American Society of Breast Surgeons (ASBrS) solicited candidate measures for the *Choosing Wisely*^®^ Campaign. The resulting list of “appropriateness” measures of care was ranked by a modified Delphi appropriateness methodology. The highest-ranked measures were submitted to and later approved by the ASBrS Board of Directors. They are listed below.

**Results:**

(1) Don’t routinely order breast magnetic resonance imaging in new breast cancer patients. (2) Don’t routinely excise all the lymph nodes beneath the arm in patients having lumpectomy for breast cancer. (3) Don’t routinely order specialized tumor gene testing in all new breast cancer patients. (4) Don’t routinely reoperate on patients with invasive cancer if the cancer is close to the edge of the excised lumpectomy tissue. (5) Don’t routinely perform a double mastectomy in patients who have a single breast with cancer.

**Conclusions:**

The ASBrS list for the *Choosing Wisely*^®^ campaign is easily accessible to breast cancer patients online. These measures provide surgeons and their patients with a starting point for shared decision making regarding potentially unnecessary testing and operations.

When surveyed, nearly three of four U.S. physicians say “doctors” order unnecessary tests and procedures. A similar proportion report that they themselves order unnecessary studies and interventions as often as once per week and that almost half of their patients request unnecessary tests on a weekly basis as well.^1^ These personal observations from “doctors” are accompanied by objective evidence of variation in the quality and value of care delivered to breast cancer patients.^2^ Both underutilization of evidence-based care and overutilization of unnecessary services have been documented.^3^–^18^ The former can lead to worse cancer outcomes, whereas the latter increases the cost of care without increasing value.^16^ To address these concerns across all specialties, the American Board of Internal Medicine (ABIM) Foundation launched an initiative to improve “appropriateness” of medical care in the United States. The *Choosing Wisely*^*®*^ campaign was launched in 2012, and more than 70 professional organizations have now contributed lists of appropriateness of care.^1^ The goals of this program are to “promote conversations between clinicians and patients by helping patients choose care that is supported by evidence and truly necessary.” The campaign empowers patients to engage their care providers in a thoughtful discussion of the benefits, risks, and effectiveness of the services offered to patients.

The inspiration for *Choosing Wisely*^*®*^ came from Howard Brody in 2010, when he challenged specialty societies to create “top 5” lists of tests and procedures that had not been shown to provide meaningful benefits to some patients for which they were ordered.^19^ Nine societies submitted lists in 2012. An increasing number have joined each year since that time. The purpose of the report described herein is to describe the American Society of Breast Surgeons (ASBrS) effort to identify, create, and endorse five measures of appropriate care.

## Methods

The ASBrS has more than 3000 surgeon and associate members.^20^ After approval from its Board of Directors, their Quality Committee (QC) solicited potential “appropriateness measures” of breast care from the general and QC membership in 2014 and 2015. The QC then corresponded with the ABIM to establish the scope and clarity of the ABIM mission. The QC (13 members) were provided with the *Choosing Wisely*^*®*^ goals and existing “choices” previously recommended by other organizations for breast cancer (Table 1).^21^ Committee members received the following instructions to rank our final list of 38 choices:

**Table 1:** Breast appropriateness measures in the *Choosing Wisely* ^®^ Campaign

Society	Recommendation
AMDA The Society for Post-Acute and Long-Term Care Medicine	Don’t recommend screening for breast, colorectal or prostate cancer if life expectancy is estimated to be less than 10 years
American Geriatrics Society	Don’t recommend screening for breast, colorectal, prostate, or lung cancer without considering life expectancy and the risks of testing, overdiagnosis, and overtreatment
American Society of Clinical Oncology	Don’t use combination chemotherapy (multiple drugs) instead of chemotherapy with one drug when treating an individual for metastatic breast cancer unless the patient needs a rapid response to relieve tumor-related symptoms
American Society of Clinical Oncology	Don’t perform surveillance testing (biomarkers) or imaging (PET, CT, and radionuclide bone scans) for asymptomatic individuals who have been treated for breast cancer with curative intent
American Society of Clinical Oncology	Don’t perform PET, CT, and radionuclide bone scans in the staging of early cancer at low risk for metastasis^a^
American Society for Radiation Oncology	Don’t initiate whole breast radiotherapy as a part of breast conservation therapy in women age ≥50 years with early-stage, invasive breast cancer without considering shorter treatment schedules
American Society for Radiation Oncology	Don’t routinely recommend follow-up mammograms more often than annually for women who have had radiotherapy following breast conserving surgery
American Society for Radiation Oncology	Don't routinely use intensity modulated radiotherapy (IMRT) to deliver whole breast radiotherapy as part of breast conservaiton therapy
American Society of Plastic Surgeons	Avoid performing routine and follow-up mammograms of reconstructed breast after mastectomies^a^
American Society of Plastic Surgeons	Avoid using drains in breast reduction mammoplasty
American Society of Plastic Surgeons	Avoid performing routine mammograms before breast surgery
American College of Surgeons	Don’t perform axillary lymph node dissection for clinical stages I and II breast cancer with clinically negative lymph nodes without attempting sentinel node biopsy^a^
Commission on Cancer	Don’t perform surgery to remove a breast lump for suspicious findings unless needle biopsy cannot be done^a^

^a^Choices that were ranked in the “highest tier” by the ASBrS but not included in the ASBrS list to avoid redundancy

1. To rank for appropriateness and value of care; value to be characterized by both quality of care and “burdens of care.”^22^,^23^
2. To rank based on the “importance” criteria of the National Quality Forum for quality measures—importance, scientific acceptability, feasibility, and usability.^24^

Two rounds of modified Delphi process ranking were performed electronically: March 2014 and July 2015.^25^,^26^ A complete Delphi process of ranking continues until all participants are in uniform agreement; our process of two rounds was therefore a “modified” Delphi process.

Each potential choice for a measure of appropriateness was ranked on a scale of 1 (no value or importance) to 9 (highest possible value or importance). After the first round, a spreadsheet of median scores was provided to committee members, allowing opportunity for participants to lobby for either increasing or decreasing a choice’s “rank.”

The final voting panel included nine QC members. Appropriateness of a measure is achieved in a panel of nine with a median score of 7–9, if there is no major “disagreement” between panelists, as defined by fewer than three panelists scoring the measure from 1 to 3.^25^ There were 16 choices deemed appropriate by this method. The top 5 choices had median ranks of 8 or 9. Four of these top 5 choices were already included in the *Choosing Wisely*^*®*^ Campaign from other organizations, based on ABIM policy, and these were excluded from our list. Their domains of appropriateness were to encourage needle biopsy as the preferred method of diagnosis, limit routine mammography of reconstructed breasts after mastectomy and discourage the use of pre- and postoperative systemic imaging in asymptomatic patients with breast cancer (Table 1). To finish our list of five, we used the next highest-ranked choices. The final list of five choices was then formatted in the style specified by the ABIM. The list was submitted to the ABIM and a manuscript was drafted and later approved by the ASBrS Research Committee and Board of Directors on April 12, 2016.

## Results

Five Tests or Interventions Physicians and Patients Should Question

1. Don’t routinely order breast magnetic resonance imaging (MRI) in new breast cancer patients.^27^–^34^
  After a new diagnosis of breast cancer, breast MRI can be useful in selected patients, to aid treatment decisions, including but not limited to those with occult breast cancer presenting with axillary metastases or patients with genetic mutations predisposing to increased breast cancer risk. However, there is a lack of evidence that routine use of MRI lessens cancer recurrence, death from cancer, or the need for reoperation after partial breast removal (lumpectomy) surgery. The routine use of MRI is associated with an increased need for subsequent breast biopsy procedures, delays in time to treatment, and higher cost of care. In addition, more patients may undergo mastectomy *with* routine use of MRI due to MRI detection of findings of uncertain significance that result in increased patient anxiety and their subsequent decision to undergo mastectomy even without proof of other cancer(s).
2. Don’t routinely excise all the lymph nodes beneath the arm in patients having partial breast removal (lumpectomy) for breast cancer when only one or two contain cancer.^28^,^35^,^36^
  After a new diagnosis of invasive breast cancer, most patients undergoing partial breast removal (lumpectomy) benefit from a “sentinel node (SN) mapping surgery”—a procedure that removes a small number of lymph nodes beneath the arm that drains the known cancer. In the past, patients found to have cancer in any SN underwent extra surgery to remove more nodes. Recent evidence suggests that further node surgery is not necessary in patients with cancer found in fewer than three SN, if the patient receives other recommended cancer treatments.
3. Don’t routinely order specialized tumor gene testing in all new breast cancer patients.^28^,^37^–^41^
  There are multiple, new, tumor “multigene signature” tests that provide selected breast cancer patients with information about their risk of distant cancer recurrence, dying of cancer, or the likelihood that they will benefit from chemotherapy. These tests are helpful in selected patients, including those with early-stage, hormone-receptor–positive cancers with “low” scores on 21 gene recurrence testing, who can safely omit chemotherapy. There is no evidence that these types of tests should be used routinely. They should not be performed in patients who indicate that the test results would not change their choice of treatment.
4. Don’t routinely reoperate on patients if the cancer is close to the edge of the excised lumpectomy tissue.^28^,^42^–^45^
  Patients undergoing partial breast removal (lumpectomy) of the breast and whole breast radiation for *invasivecancer* benefit from reoperation to excise more breast tissue if microscopic review of the lumpectomy breast tissue indicates that cancer cells are present at the tissue edge. However, if cancer cells are close to the edge, but not at the actual edge, then recent evidence indicates that reoperation is not mandatory.
5. Don’t routinely perform a double mastectomy in patients who have a single breast with cancer.^46^–^53^
  After a new diagnosis of breast cancer in a single breast, many patients desire removal of both breasts, believing their cancer risk in the other breast is high and their cancer cure rate will be improved with double mastectomy. Double mastectomy should not be routinely performed in average-risk patients until they have been provided with adequate understandable information about the generally low risk that they will develop cancer in the other breast and the minimal effectiveness, if any, of double mastectomy to improve their life expectancy or survival from breast cancer.

## Discussion

The *Choosing Wisely*^*®*^ campaign was launched to advance the patient-provider dialogue such that unnecessary medical tests, treatments, and procedures would be used less often.^1^ To accomplish this, the ABIM recommended that professional organizations provide the ABIM with “five things providers and patients should question.” 1 Conceptually, the provider stakeholders create lists of domains of care decisions intended to spur conversations between providers, patients, and payers about appropriate care, resulting in less “waste.”^54^,^55^ This process is in alignment with widely accepted principles to increase the “value” of healthcare, by lowering cost, promoting patient engagement, and creating a result that is measurable.^56^ If initiatives to increase adherence to *Choosing Wisely*^*®*^ choices are successful, then the national cost of healthcare is likely to decrease. In a cohort of 22,000 patients enrolled in a single insurance plan in the state of Washington, an estimated cost savings of $29 million was achieved through increased adherence to five *Choosing Wisely*^*®*^ choices.^10^

After first recognizing and then taking ownership of the effort to reduce overutilization of services, the ASBrS developed a list of measures that were intended to improve appropriateness of testing and surgery in patients with breast cancer. Measures were chosen that were deemed important by the criteria of the NQF for quality measure development.^24^ These criteria included but were not limited to scientific support, evidence of variability of care, and feasibility of use. In the ABIM campaign, all measure choices should be usable, because they only require the provider to discuss the measure with the patient. Patient use and understanding of the *Choosing Wisely*^*®*^ choices also is facilitated by the ABIM’s required formatting to include simple, understandable, and brief declarative statements, usually beginning the statement with “Don’t.”

Other organizations that care for patients with breast cancer have submitted their *Choosing Wisely*^*®*^ Lists to the ABIM (Table 1).^21^ All used different methods for developing and prioritizing their lists. None used our modified Delphi ranking process; yet, independent of these other organizations, 4 of our 5 top choices were already selected by them. This concordance between developers using different methodologies supports the importance and potential impact of these specific measures.

A systematic review of the literature for each of our *Choosing Wisely*^*®*^ choices is not the intent of this report. Background information, comprehensive reviews, and evidence-based support for each of our measures is referenced. It is important to note that our “choices” are not meant to infer that the test or procedure endorsed in our list is a “never should occur” event akin to “wrong site” surgery. For example, we recommend against “routine MRI” in new breast cancer patients, but MRI imaging can be useful in selected patients to aid treatment decisions, including but not limited to those with occult breast cancer presenting with Paget’s disease of the nipple or with axillary metastases or patients with mutations predisposing to increased breast cancer risk.^27^ Rather, the services listed should be discussed with patients and shared decision making should occur. There may be circumstances in which best, highest-quality care is different from the *Choosing Wisely*^*®*^ statement on a particular topic. Moreover, there are no benchmarks established for what level of compliance with our statements would be desirable. Three of our “choices”—on MRI usage, SN surgery, and margin status decisions—have a high level of evidence supporting them based on randomized, controlled trials and/or meta-analyses.^27^–^36^,^42^–^45^ For the other two choices—contralateral prophylactic mastectomy (CPM) and tumor multigene signature testing—there is evidence of increasing utilization that is not always accompanied by evidence of improving patient-reported and/or clinical outcomes.^28^,^37^–^41^,^46^–^53^

CPM rates have increased during the past decade.^46^–^49^ In average-risk patients, there is a lack of convincing evidence that CPM improves cancer-specific survival. These operations often are driven by patient requests for risk reduction or symmetry. Many patients with unilateral breast cancer request a CPM, because they perceive that their cancer risk in the other breast is higher than their actual risk.^51^ The inclusion of CPM in the ASBrS choices for *Choosing Wisely*^*®*^ does not mean that the ASBrS endorses a policy of never performing it. We are simply recommending full education regarding its risks and benefits, emphasizing the importance of a decision-making process shared by patients and providers. A full discussion of CPM, its indications and contraindications, is beyond the scope of this report, but the ASBrS held a consensus conference in 2016 to further characterize the reasons to consider or discourage CPM (J. Boughey, Program Director for the 2016 Annual Meeting of the American Society of Breast Surgeons, personal communication, April 12, 2016).

Tumor multigene signature panels that are prognostic for risk of distant recurrence and overall survival and predictive of benefit of chemotherapy are increasingly utilized after new breast cancers are diagnosed.^39^–^41^ In select patients with invasive cancer, the use of a “validated” tumor multigene signature testing panel is appropriate if the patient’s tumor characteristics were consistent with those used in the panel validation studies and if the results of testing would affect the patient’s decisions regarding adjuvant treatment. In this scenario, the test identifies patients in whom chemotherapy can be omitted, without harm to distant recurrence risk or overall survival.^39^,^41^ New and emerging tumor multigene panels hope to do the same, but not all have yet been validated. Panels also are available for patients with ductal carcinoma in situ.^57^ Industry, patients, and patients’ families may pressure surgeons to order these tests to help direct therapy. As a result, there is risk of overutilization of testing without concomitant patient benefit if validated tests are ordered for patient subgroups not included in the validation studies or if a patient has already decided to omit a specific adjuvant therapy due to age, comorbidities, or personal reasons.

The *Choosing Wisely*^*®*^ campaign is still in its relative infancy, having been in existence for less than 6 years. Enthusiasm for its potential impact on improving appropriate care and reducing waste is evidenced by the submission of appropriateness measures by more than 70 professional societies, increasing organizational participation each year, and the rapid emergence of research projects and publications measuring adherence to the *Choosing Wisely*^*®*^ choices. Many organizations and regional quality collaboratives are now auditing compliance with the *Choosing Wisely*^*®*^ choices, using them as a surrogate measure of quality.^58^ Most *Choosing Wisely*^*®*^ choices also could be crafted into metrics of value or efficiency. Although results are preliminary, some organizations have already implemented action plans to address compliance variability.^59^

We hope that our society’s endorsement of five new choices for the *Choosing Wisely*^*®*^ campaign will contribute to increased delivery of appropriate care and decreased overall cost of care for breast cancer patients. Plans to develop five additional measures of appropriateness in the management of benign breast disease are anticipated.
